# Student Children

**Published:** 1870-08

**Authors:** 


### STUDENT CHILDREN.

No child ought to be sent to school to study until seven years old.

Until twelve, no child should be kept in the school-room over two hours at a time. Four hours a day at study is enough for a child under twelve years of age. To compel any child under twelve to sit or stand perfectly still for ten minutes is a barbarism; because it is the motion of the muscles which aid to send the blood to the heart, and if they are quiet too long, the blood goes to the heart in too small quantities to keep it in action, hence it stops beating, and the person is said to "faint." So necessary is motion to the circulation of the blood, that even in our sleep, Nature's ever watchful instincts cause a change of position every few moments either in the body or some of the limbs.

NIGHTMARE

is caused by remaining so long in one position that the blood ceases to circulate. How hard we try to run in our sleep sometimes to get out of the way of some terrible danger; it does such a person no good to ask what's the matter; don't waste time in asking a question, but give relief to the sleeper by an instantaneous shake, or even a touch of the body, that breaks the dreadful spell in an instant, because it sets the blood going again towards the heart.

